# The effect of varicocele on semen quality in boars exposed to heat stress^1^

**DOI:** 10.1093/tas/txaa003

**Published:** 2020-01-17

**Authors:** Tasha R Gruhot, Lea A Rempel, Brett R White, Benny E Mote

**Affiliations:** 1 Department of Animal Science, University of Nebraska–Lincoln, Lincoln, NE; 2 United States Department of Agriculture, Agricultural Research Service, United States Meat Animal Research Center, Clay Center, NE

**Keywords:** boars, fertility, heat stress, semen quality, varicocele

## Abstract

Semen quality has a dramatic impact on reproductive efficiency in the swine industry, influencing both conception rate and litter size. The objective of this study was to assess whether the presence of varicocele hinders semen quality in both thermoneutral and heat stress (HS) conditions. At approximately 6 mo of age, ultrasonography was used to measure left and right pampiniform plexus area in order to detect varicocele in maternal line boars at the University of Nebraska–Lincoln. Between 10 and 12 mo of age, semen was collected from each boar (*n* = 28) twice weekly. Boars were collected under thermoneutral conditions, were then heat stressed for 7 d to exacerbate any semen quality issues, and semen was collected post-HS for 6 wk. Sperm characteristics were determined by computer-assisted semen analysis. The presence of varicocele had a significant effect on sperm concentration (*P =* 0.04) and trended toward significance for mean sperm head area (*P =* 0.06) throughout the duration of the study. An interaction existed between varicocele and collection time point at weeks 2–5 post-HS for distal droplet percentage, suggesting that boars with varicocele were possibly more susceptible to heat-stress-induced semen quality issues than boars without varicocele. Moreover, semen quality was reduced in boars with versus without varicocele under both thermoneutral and HS conditions. Therefore, detection of varicocele by ultrasound could represent a potential marker of fertility in young boars or as a component trait in selection indices for fertility.

## INTRODUCTION

Prior to the use of a boar in matings, a semen quality assessment is conducted on the ejaculate as semen quality has a significant impact on economically important traits, such as conception rate and litter size (Waberski et al., 1994; Rozeboom, 2000). The rejection rate of boar ejaculates is increased during the summer months due to decreased semen quality resulting from heat stress (HS; Flowers, 1997; Stone, 1982; Boyer and Almond, 2017). Impaired spermatogenesis is reported to occur at temperatures as low as 22.2 °C, which is only slightly above the thermoneutral (TN) zone (17.7–20 °C) of boars (Blackshaw, 1977; Stone, 1977; Boyer and Almond, 2017).

Physiological factors also play a role in semen quality and fertility. For example, vascular abnormalities, such as dilated tortuous veins and/or vascular lesions of the pampiniform plexus (PP), referred to as varicocele, have been associated with male infertility (Jarow, 2001). When varicocele is present, the abdominal blood flowing to the testes is not properly cooled due to hindered countercurrent heat exchange, resulting in increased testicular temperatures and impaired testicular function (Hsiung et al., 1991). In men with infertility issues and reduced semen parameters, varicocele is the most commonly reported physical abnormality (Sigman and Jarow, 1997; Sigman and Howards, 1998). The presence of varicocele in boars has a significant effect on sperm membrane integrity and is associated with increased morphological abnormalities (Kleve-Feld et al., 2015). A prevalence of approximately 23% of boars has been reported in several boar populations under different methods of diagnoses: palpation in adult boars (Kleve-Fled et al., 2015), ultrasonography in young boars (Gruhot et al., 2019), and during necropsy in adult boars culled due to poor semen quality (Ubeda et al., 2014). Therefore, the objective of the current study was to evaluate boars for the presence of varicocele by ultrasound and determine the relationship of varicocele with specific computer-assisted semen analysis (CASA) semen quality traits under both TN and heat-stressed conditions.

## MATERIALS AND METHODS

Research protocols and methods were approved by the Institutional Animal Care and Use Committee at the University of Nebraska–Lincoln (UNL).

### Boars and Varicocele Determination

All intact boars from generation 35 of the Nebraska Index Line (Petry and Johnson, 2004) (*n* = 127) housed at the UNL Swine Research Farm were assessed for the presence of varicocele. Boars were evaluated via ultrasonography to estimate average PP vessel size at 199 ± 6 d of age. Ultrasonography was performed on the PP for each testicle, and the average area of the right (AAR) and left (AAL) PP vessels was derived, following the ultrasonography protocol and method of estimating the PP area described by Gruhot et al., (2019). A coronal–saggital image of the PP for each testicle was acquired using ultrasonography with a linear endorectal probe (Ibex EVO, E.I. Medical Imaging, Loveland, CO). The probe was held at a 45° angle on the outer lower portion of the testicle. The Bioquant collage image system (Bioquant Image Analysis Corp., Nashville, TN) was used to measure the area (mm^2^) of 3–10 vessels of the PP. The AAR and AAL were derived, as well as the standard deviation of the PP vessels for each testicle. To evaluate if a boar was suspected of having varicocele, a within-boar vesicle ratio was estimated for each testicle. The within-boar ratios were estimated as: (AAR of boar X/AAL of boar X) and (AAL of boar X/AAR of boar X). If the boar’s average vessel area was at least 1.5 times larger than the opposing average plexus vessel area, those vessels were considered suspect varicocele. Varicocele status, presence of varicocele (VP) or absence of varicocele (VA), was treated as a binary trait for each boar.

From this group, 28 boars were utilized for semen evaluation based on their classification of VP or VA. Thirteen of the boars were considered VP and 15 boars were classified as VA. The 28 boars were randomly placed into one of two HS trials, in which trial 1 contained seven boars with VP and seven boars with VA and trial 2 contained six boars with VP and eight boars with VA.

### HS and Semen Collection Trials

The boars (*n* = 28) were trained for semen collection from a phantom mount and put on a twice per week collection schedule, with boars being collected on a Monday/Thursday or Tuesday/Friday. Boars were collected for 3 wk on this schedule prior to being included in the study. Two collections were processed in the week prior to the boars being heat stressed and these were considered the TN collection time points for data comparison. Boars entered the HS barn on a Wednesday and exited the following Wednesday (7 d). Post-HS collection began the Thursday/Friday following the return of boars to normal TN housing, with this collection considered the week 1 post-HS collection. For weeks 2–6, the boars were collected twice weekly following their previous collection schedule. Due to the distance between the HS barn and the collection barn, boars were not collected while being subjected to HS. Two collection trials were conducted due to limited pens in the HS barn (*n* = 14) and time/labor required to collect and process semen.

The first trial (trial 1) was conducted (mid-January 2017) using 14 boars that were approximately 10 mo of age at the start of the TN collections. The second trial (mid-March 2017), which also utilized 14 boars, consisted of boars that were approximately 12 mo of age at the start of the TN collections.

During the HS week (7 d), the boars were subjected to a minimum temperature of 29.4 °C during the day for 9 h; the temperature was then lowered to 23.9 °C overnight. Outside temperatures never exceeded barn temperatures during the HS trials. Ad libitum access to water was provided at all times. Boars were fed a total of 1.8 kg of a fortified corn and soybean meal-based diet daily. Feeding occurred twice a day at approximately 700 and 1400 hours. Boars were housed in individual pens in the HS barn and were kept in individual stalls in the collection barn.

### Semen Analysis

Semen was processed using the Hamilton–Thorne Sperm Analyzer IVOS 1.9 CASA system (Hamilton Thorne Biosciences, Beverly, MA). A 20-μL aliquot of semen was evaluated using a Leja4 analysis chamber (Leja, Nieuw-Vannep, Netherlands) at 35 °C. Semen quality traits assessed via CASA were the following: total percent motile (MOT), percent progressively motile (PROG), distal droplet percent of total (DIST), bent tail percentage (BENT), proximal droplet percent of total (PROX), total concentration (billion per milliliter; CONC), total sperm cells in ejaculate (billion; TOTSP), and total mean sperm head area (AREA) measured in square microns. Ejaculate volume (VOL) was also analyzed by weight in grams.

### Statistical Analysis

Statistical analysis was performed in R (R Core Team, 2017). A Welch two-sample *t*-test was used to test for a significant difference in the means of average PP vessel size between boars with and without varicocele. Normality of the data was assessed using the Shapiro–Wilk test of normality. If the distribution of the variable was significantly different from a normal distribution, log or square root transformations were performed. Log transformations were performed on: BENT, PROX, and DIST; and square root transformations were performed on: CONC, TOTSP, and VOL. Using the “lme4” package in R, the following linear models were utilized to estimate regression coefficient estimates of the various semen parameters (i.e., MOT, PROG, DIST, PROX, BENT, CONC, TOTSP, AREA, and VOL) on the fixed effects in the model. Fixed effects in the models included: presence of varicocele (VA or VP), trial, collection time point (week), and week by presence of varicocele (VA or VP) interaction. The interaction was included to assess if boars with varicocele handled HS differently than boars without varicocele. Interactions with trial were tested for, of which none were significant and, thus, no trial interactions were included in the model. A random effect of animal was included to account for repeated records on the boars. A repeated-measures analysis of variance (ANOVA) type III SS (from the Car package) was used to test the effects in the model. Least squares (LS) means were estimated using the Emmeans package. If the semen quality trait had been transformed, LS means estimates were back-transformed to be reported on their original scale.

## RESULTS

The boars classified as having varicocele (predominantly left-side varicocele) had an AAR of 5.88 ± 2.88 and an AAL of 10.1 ± 3.89. The boars not considered to have varicocele had an AAR of 8.26 ± 2.76 and an AAL of 8.66 ± 3.02. These differences in PP size between VP versus VA boars were significantly different: AAR (*P* < 0.001), AAL (*P* = 0.02).

None of the models showed a significant interaction between presence of varicocele and week. The lack of a significant interaction between varicocele presence and collection time point indicates that there was no statistical difference in the boar’s ability to handle HS based on varicocele status. However, the interaction of varicocele status and week trended toward significance for DIST (*P* = 0.06). This trending interaction was observed in the regression coefficient estimates for presence of varicocele by week, being statistically different from 0 (*P* < 0.05) for week 2: collection 1 through week 5: collection 1. However, in the pairwise contrasts of presence of varicocele by week, only week 3: collection 1 showed the means of VA and VP to be statistically different (*P* = 0.03). The week 3: collection 1 LS means were as follows: VA = 6.55 ± 0.74, VP = 8.88 ± 0.80, resulting in a significant 2.33% ± 1.08 difference in distal droplets, indicating VP boars had a larger increase in distal droplet percentage at this post-HS collection time point than VA boars.

A significant difference between the means of VA and VP was found for CONC (*P* = 0.04) with the VA boars having an additional 0.03 billion more sperm cells per milliliter in the ejaculate than the VP boars. Total head area tended to be larger in VA than VP boars (*P* = 0.06) at 17.63 µm^2^ and 17.22 µm^2^, respectively. Varicocele status had no significant effect on MOT, PROG, PROX, BENT, TOTSP, and VOL. Estimated LS means by varicocele group (VA or VP) for all semen quality traits are shown in Table 1.

**Table 1.** Least squares means estimates (±SEM) of semen quality parameters by varicocele status

Semen trait	VA^*a*^	VP^*a*^
Motility, %	77.78 ± 3.30	77.76 ± 3.55
Progressive motility, %	66.84 ± 3.10	62.36 ± 3.38
Distal droplet, %	6.03 ± 0.22*	7.12 ± 0.31*
Proximal droplet, %	4.89 ± 0.59	5.63 ± 0.55
Bent tail, %	3.75 ± 0.26	3.78 ± 0.29
Concentration, billion/mL	0.12 ± 0.00*	0.09 ± 0.00*
Total sperm per ejaculate, billion	30.92 ± 2.18	26.83 ± 2.19
Head area, square microns	17.63 ± 0.15^*b*^	17.22 ± 0.22^*b*^
Volume, grams	286.29 ± 14.58	314.89 ± 16.70

^*a*^Varicocele absent (VA) or varicocele present (VP).

^*b*^Means for VA and VP tended to be different (*P* = 0.06).

*Means for VA and VP statistically were different (*P* < 0.05).

## DISCUSSION

The results of the current study indicated that varicocele had a negative impact on sperm concentration. Although not significant, total sperm produced per ejaculate was numerically greater in VA boars, whereas total ejaculate volume was numerically greater in VP boars. This relationship can explain why VA boars had a greater concentration per milliliter as a lesser volume with greater sperm output would lead to a greater concentration. In humans, similar results have been reported. Men with varicocele were found to have lesser sperm concentration than men without varicocele, regardless of fertility status (Pasqualotto et al., 2005).

Infertile men with varicocele had greater than normal circulating concentrations of follicle-stimulating hormone (FSH) and reduced sperm concentration. It was suggested that this increase in FSH might reduce sperm concentration via an alteration of Sertoli cell function (Cayan et al., 1999; Pasqualotto et al., 2005). Other studies have reported decreased testosterone biosynthesis in men with varicocele caused by a disruption in Leydig cell function (Cayan et al., 1999). Intratesticular hyperthermia is likely influencing these hormonal differences as it has been shown that varicocele increases testicular temperatures (Hsiung et al., 1991). Proper Leydig and Sertoli cell functions need to occur within the testis for normal spermatogenesis to take place, and any hindering of these cell functions can be detrimental to the spermatogenesis process (Singh, 2016). Though these findings have not been confirmed in the boar, it is possible that these same biological alterations shown in humans due to varicocele could be causing the difference in concentration between the boar groups in the current study.

An increase in morphologically abnormal sperm has been previously reported in boars with varicocele (Kleve-Feld et al., 2015). The current study also observed a greater rate of distal droplets in boars with varicocele compared to boars without varicocele. Distal droplets occur more commonly than proximal droplets in boar ejaculates and have a more severe impact on production when compared to proximal droplets (Waberski et al., 1994; Rozeboom, 2000). The extended retention of distal droplets following ejaculation has been associated with infertility in boars (Waberski et al., 1994; Kuster et al., 2004) and bulls (Amann et al., 2000; Thundathil et al., 2001). Specifically, a negative correlation between distal droplets percentage with conception rate, and litter size has been reported (Waberski et al., 1994).

Increased distal droplet percentage after HS has been well documented (Cameron and Blackshaw, 1980; Stone, 1982). Collection time point week 2: collection 1 through week 5: collection 2 showed interactions with varicocele status as indicated by their regression coefficient solutions being statistically different than 0. In addition, the means for VA and VP were statistically different at week 3. These post-HS collection time points are when effects from HS are expected based on the boar’s spermatogenesis cycle as the critical period of time for proper spermatozoa development is 19–33 d prior to semen collection (Gibbs et al., 2013). The interaction of varicocele within this time period suggests that varicocele is potentially influencing how the boar’s testicles are able to handle HS. Thus, it is possible that boars with varicocele may be at an increased risk for semen quality issues when exposed to HS, though confirmation in additional populations is needed.

Proper sperm head function is vital as the sperm must undergo capacitation and the acrosome reaction, as well as bind to the zona pellucida and plasma membrane of the oocyte for successful fertilization (Kaskar et al., 1994). In humans, varicocele has been shown to be associated with head defects of spermatozoa, increasing the number of tapered and amorphous heads, and spermatozoa with head defects have been associated with decreased pregnancy rates (Schatte et al., 1998). In the current study, boars with varicocele had smaller average sperm head area than boars without varicocele. A recent study published by Mandawala et al., (2018) reported that higher mean nucleus area in boars, which is a function of greater head width and lower variability between sperm cell heads, positively influenced fertility. Previous work by Rempel et al., (2019) identified that boars with reduced head shape from spring to summer subsequently had reduced fertility rates in comparison to boars with similar head shape at spring or summer collections.

A difference in motility between boars with or without varicocele was not observed. Consistent with this, previous assessments of motility in boars with varicocele (Kleve-Feld et al., 2015) did not differ from boars without varicocele. In humans, significantly decreased sperm motility has been observed in men with varicocele (Cayan et al., 1999; Kamal et al., 2001; Pasqualotto et al., 2005). However, human and boar spermatozoa have different tail lengths. In humans, the spermatozoa tail is much longer than boars (Cummins and Woodall, 1985) and this may explain the discrepancy in motility between species, suggesting that varicocele may impact tail development and/or function, thereby altering motility.

## CONCLUSION

Boars without varicocele appear to have maintained better semen quality under both TN and poststress conditions. These results indicate that boars with varicocele may be at risk for lower conception rates based on reduced semen quality and would service less sows due to reduction in concentration. As this study was conducted in a single population, with a relatively small number of boars, repetition in other populations with larger boar numbers would be valuable to confirm results. The use of ultrasound to identify boars with varicocele was shown to provide early detection to identify boars that may be at increased susceptibility to heat intolerance and impaired semen quality.
